# Supplementary material for: eIF2B localization and its regulation during the integrated stress response is cell-type specific
Source: iScience. 2024 Aug 30;27(9):110851. doi: 10.1016/j.isci.2024.110851 (PMC11414691; doi:10.1016/j.isci.2024.110851)
Supplement: Document S1. Figures S1‒S4 [file mmc1.pdf]

iScience, Volume *27*

Supplemental information

eIF2B localization and its regulation during the integrated stress response is cell-type specific

Filipe M. Hanson, Madalena I. Ribeiro de Oliveira, Alison K. Cross, K. Elizabeth Allen, and Susan G. Campbell

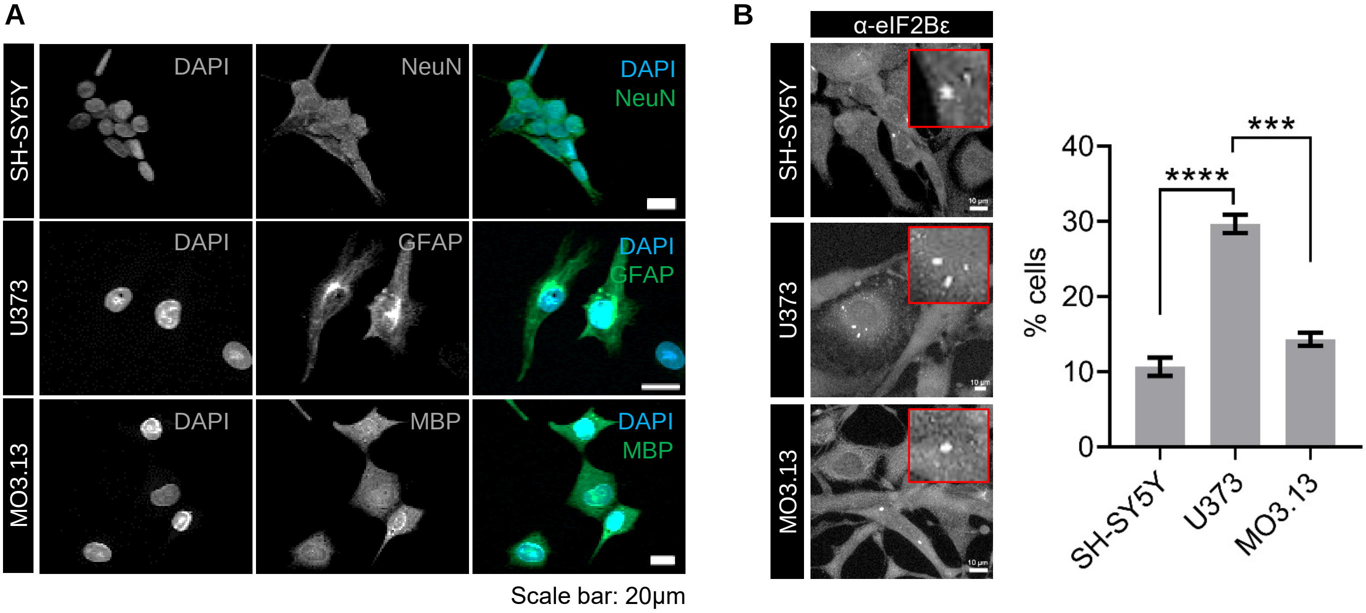

Figure S1. Endogenous eIF2B bodies are expressed in SH-SY5Y, U373 and MO3.13 cells, related to Figure 1. (A) Confocal images of SH-SY5Y, U373 and MO3.13 cells immunostained for neural marker *NeuN*, astrocytic marker *GFAP* and oligodendrocytic marker *MBP*, respectively. **(B)** Confocal images of SH-SY5Y, U373 and MO3.13 cells immunostained with α-eIF2Bε (*left panel*). Mean percentage of cells displaying at least one eIF2Bε-containing body in a population of 100 cells (mean±SEM*; N*=3; *****p*≤0.0001, ****p*≤0.001 according to one-way ANOVA) (*right panel*). Scale bar: 10 µm.

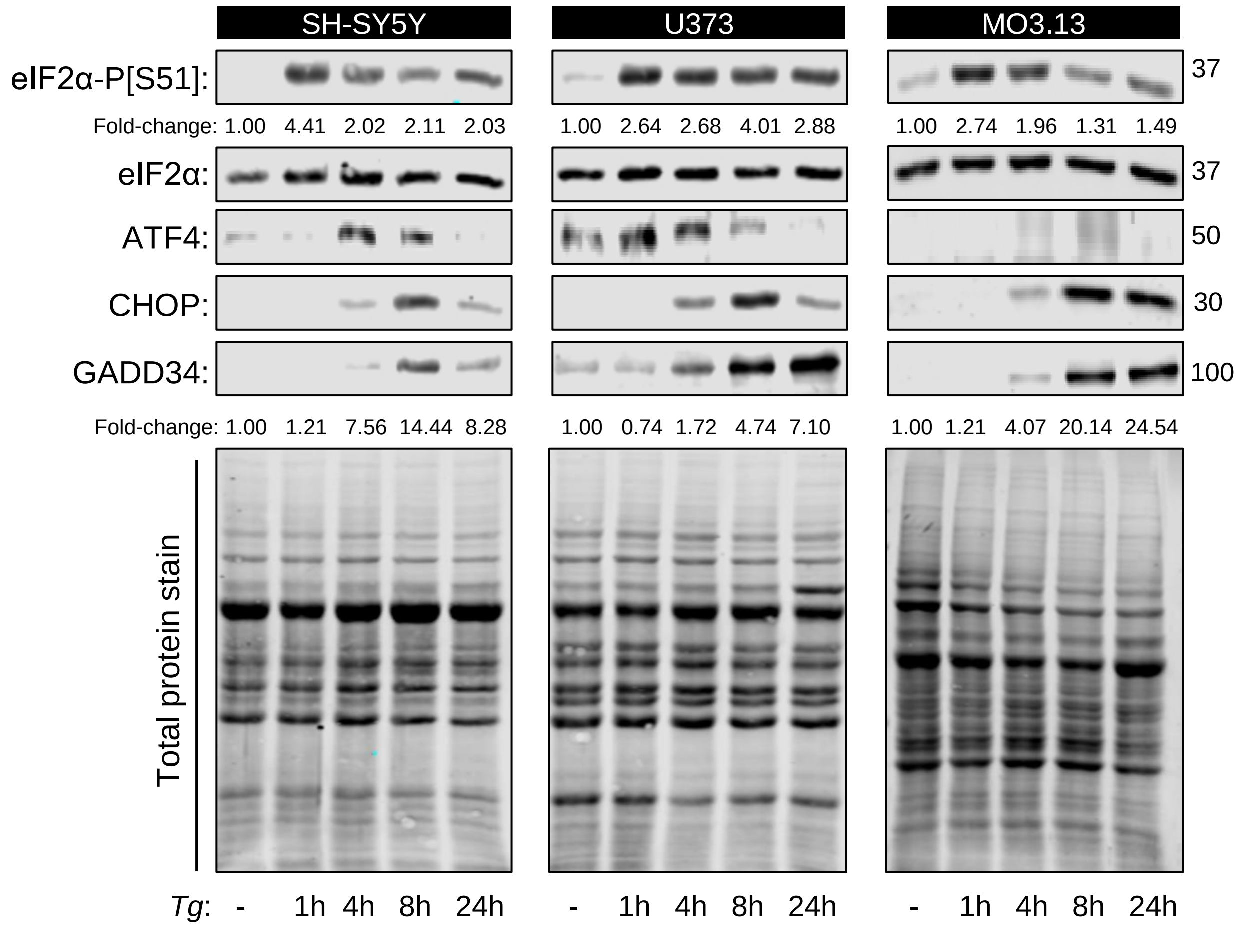

Figure S2. ATF4 expression peaks before the 24h time point of Tg exposure, related to Figure 3. Representative western blot of the ISR expression profile (eIF2α-P[S51], pan-eIF2α, ATF4, CHOP and GADD34) and total protein staining (loading control) in SH-SY5Y, U373 and MO3.13 cells treated with vehicle (DMSO) for 24h or thapsigargin for 1, 4, 8 and 24 hours.

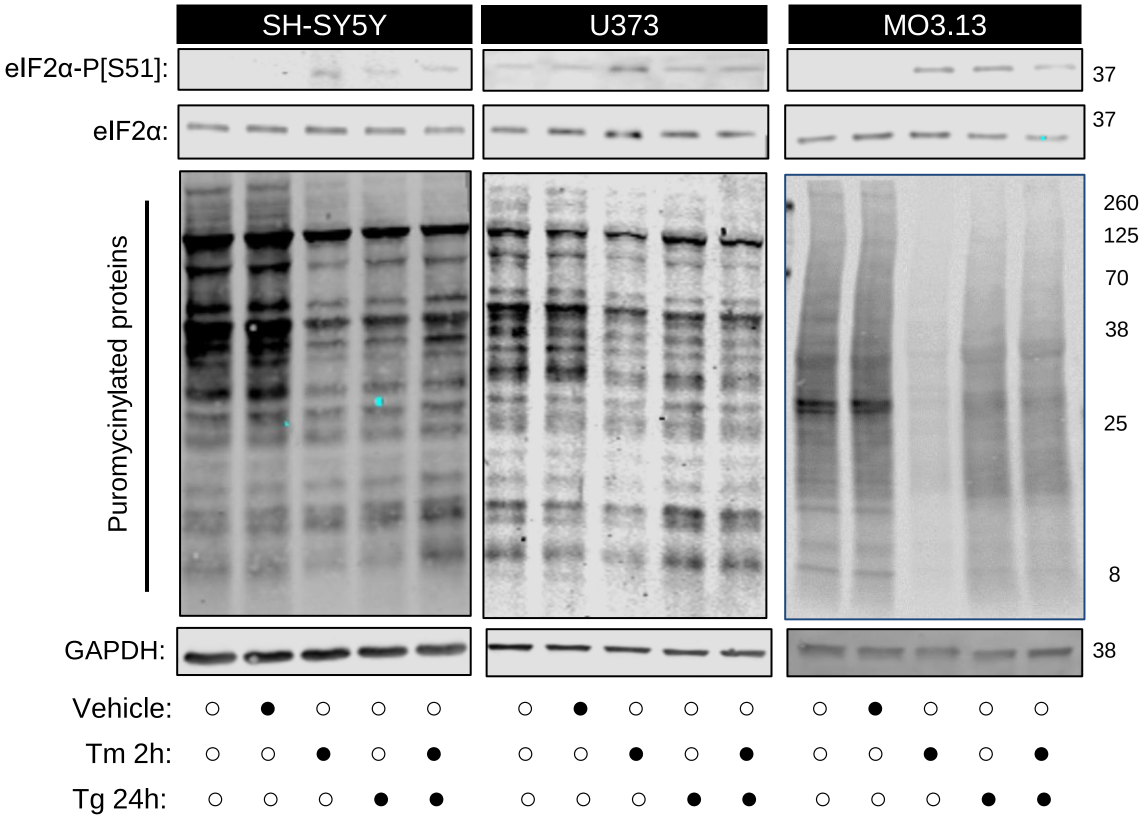

Figure S3. Tunicamycin treatment prior to Tg preconditioning for 24h does not further impact on eIF2α-P[S51] levels and protein synthesis, related to Figure 3. Western blot of the ISR expression profile (eIF2α-P[S51], pan-eIF2α) and global newly synthesized proteins (puromycin incorporation assay) in SH-SY5Y, U373 and MO3.13 cells treated with vehicle (DMSO), acute ER stress inducer (Tm 3 µg/mL for 2h), chronic ER stress (Tg 300 nM for 24h) and chronic ER stress further challenged with acute ER stress inducer (Tg 300 nM for 24h + Tm 3 µg/mL on the last 2h).

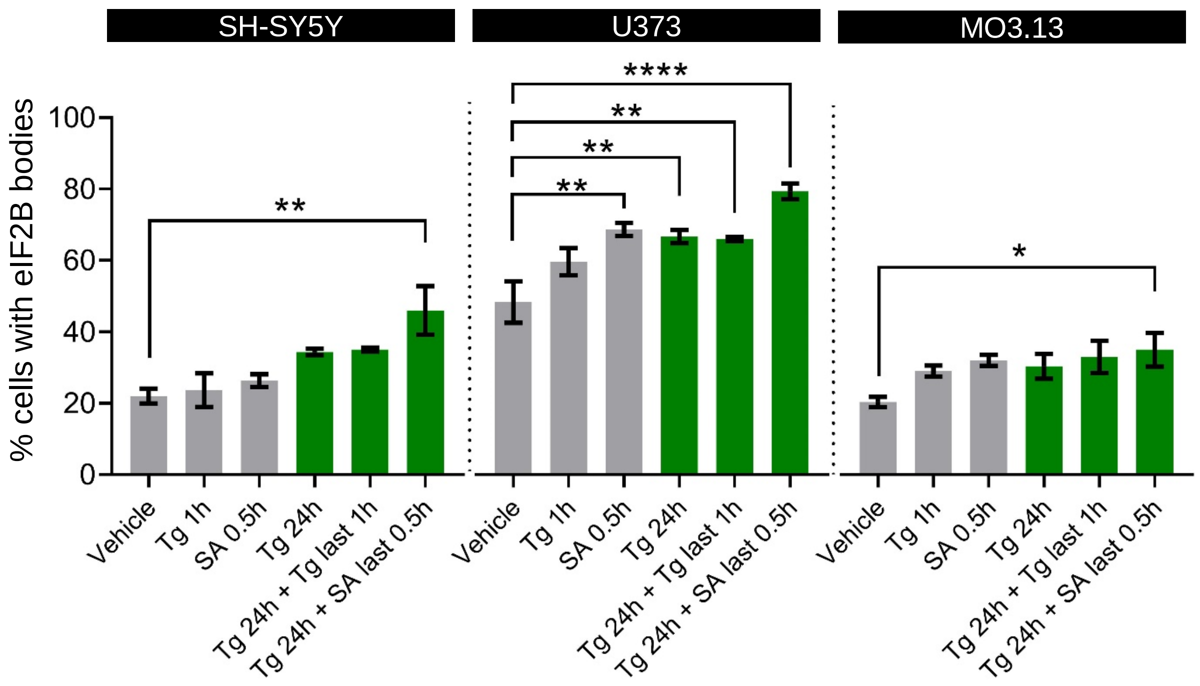

Figure S4. eIF2B localisation increases with cellular stress, related to Figure 4. SH-SY5Y, U373 and MO3.13 cells were subjected to acute stress inducers (Tg 1 µM for 1h and SA 125 µM for 30 min); chronic ER stress (Tg 300nM for 24h) or subsequently challenged with previously described acute stress treatments (Tg 24h + Tg last 1h; Tg 24h + SA last 0.5h). Mean percentage of cells displaying localised eIF2B (at least one eIF2B body present) in a population of 100 transfected cells (mean±SEM; *N*=3).
